# Saadat Hasan Manto, Partition, and Mental Illness through the Lens of *Toba Tek Singh*

**DOI:** 10.1007/s10912-019-09590-w

**Published:** 2019-12-20

**Authors:** Tahir Jokinen, Shershah Assadullah

**Affiliations:** 1 GKT School of Medical Education, King’s College London, Strand, WC2R 2LS, London, UK; 2 Lewisham and Greenwich NHS Trust, Queen Elizabeth Hospital, Stadium Road, Woolwich, London, SE18 4QH UK

**Keywords:** Partition, Literature, Mental illness, Addiction

## Abstract

“Toba Tek Singh,” which describes the exchange of mental asylum inmates between India and Pakistan in the wake of partition, was perhaps Saadat Hasan Manto’s most well-known short story. Manto’s work was coloured by his experience of mental illness, including alcohol addiction and possible depressive disorder. This essay attempts to use “Toba Tek Singh” as a lens through which to shine an integrative light on the role of mental illness in Manto’s work and life, by discussing his personal experiences, themes of mental illness in the story, and the implications of his writing in the historical context of post-partition South Asia.

## Introduction

Saadat Hasan Manto (1912-1955) was among the most famous, provocative, and controversial Urdu writers of the twentieth century. Although he was a prolific writer of essays, plays, film scripts, and a novel, he was best known for his short stories. Born in Punjab, his writing career really took off in Bombay; he then moved to Lahore in Pakistan in January 1948, some months after partition (Hasan 1984). His subsequent stories largely focused on the theme of partition, exploring its human consequences from a range of different angles. Manto’s stories are known for his tradition of realism, verbal economy, reliance on internal elements, and especially his sudden, sometimes disturbingly uncertain endings (Akhtar and Flemming 1985).

“Toba Tek Singh,” written in 1954, is perhaps Manto’s most famous short story (Jalal 2013). Describing the exchange of inmates of a Lahore mental asylum after partition, “Toba Tek Singh” uses the madness of the inmates as a mirror for the madness of the outside world. As the story progresses, the reader comes to realise that the asylum inmates are in fact much more sane than the politicians controlling their destiny. The main character, Bishan Singh, in his painful struggle for identity, is symbolic of the displacement suffered by millions of partition refugees. Although the story is fictional, an actual exchange of psychiatric patients between mental hospitals in Lahore and Amritsar took place in 1950 (Jain and Sarin 2012). Despite extensive studies of Manto’s life and work, as well as selected articles on his psychology and mental health, few attempts have been made to integrate both aspects. “Toba Tek Singh” provides a useful lens through which to look at Manto more broadly and explore the role of mental illness not only in his life and work but also in the Indian subcontinent more generally around the time of partition. By discussing Manto’s personal experience of mental illness, themes in “Toba Tek Singh” and their implications in specific historical context, this essay aims to shine an integrative light on aspects of Manto’s work and mental illness.

## Manto and mental illness

It has been established that Manto suffered mental health problems, which would undoubtedly have coloured his work. Manto’s childhood was characterised by a difficult relationship with his father who died when Manto was eighteen years old. Authoritarian and frequently belittling, Manto was afraid of his father, even on occasion jumping from the rooftop to escape him (Hashmi 2012). Manto’s father’s first wife was ‘prone to fits of mental instability’ (Jalal 2013). It is conceivable that this might have been in part a consequence of the stress caused by living with his father, and Manto would have also been exposed to similar stress. Alternatively, this mental instability may have caused disruption in the domestic environment, which would itself have been a source of stress for Manto growing up. His mother, who was the second wife, was shown contempt by Manto’s paternal family, which, according to Ayesha Jalal, left a deep emotional scar (2013).

Regardless, Manto’s difficult childhood left him with memories of neglect and rejection, and he became prone to excessive displays of emotion (Jalal 2013). Restlessness and agitation troubled Manto throughout his life. Referring to the years following his father’s death, he wrote: ‘In those days of vagrancy, I felt constantly dissatisfied. A strange restlessness gripped my heart and mind...that disquietude would not go away’ (Hashmi and Aftab 2013, 1096). Ali Madeeh Hashmi has argued that this was in part due to Manto’s Existentialist philosophy. Restlessness and agitation necessarily followed his awareness that the ultimate culmination of life is death, and all human relationships and affections are therefore temporary (Hashmi 2012). This might be overstating the case, however, as Manto’s thought was also influenced by his Islamic faith, even if he was not especially devout in his adherence to Islamic norms (Jalal 2013). It has also been suggested by Hashmi and Aftab that Manto might have suffered from a depressive disorder (2013). Multiple studies have identified a higher prevalence of mood disorders in creative writers compared to the general population (Hashmi and Aftab 2013). This is certainly plausible, and there are records of Manto describing his depressed mental state, even referring to suicide on occasion. Jalal has described how, in reference to his sister’s miscarriage, ‘recalling his emotional and mental torment at the time, Manto confessed that if he had been stronger willed, he would almost certainly have committed suicide’ (2013, 53). Whether this amounted to a clinically definable suicidal ideation is unclear; nonetheless it adds weight to the hypothesis of depressive illness plaguing Manto throughout his life.

Alcohol addiction stands out as perhaps the most significant of Manto’s mental health difficulties. Although he always drank heavily, Manto’s drinking escalated after his move to Lahore. His family became so concerned that they twice admitted him to the antialcoholic ward of the Punjab Mental Hospital for treatment between 1951 and 1952 (Jalal 2013). This was not successful; Manto progressed to binge drinking and started to experience hallucinations (Jalal 2013), a symptom of alcohol-induced psychosis (Hashmi and Aftab 2013). Noting the high rates of comorbidity of alcohol addiction and mood disorders, Hashmi and Aftab have suggested that Manto used alcohol for self-medication, arguing that he ‘sought refuge in substance abuse to ease his psychological pain’ (2013, 1096). This was a time when Manto was at his lowest, struggling financially and finding it difficult to situate his sense of identity in Pakistan. Therefore, it does not seem unreasonable to associate his alcohol use with a potential depressive disorder. Ultimately, it was liver cirrhosis due to alcoholism that killed Manto at the premature age of forty-four.

Hashmi has argued that Manto’s burst of creative output was a sign that he was preparing for impending death (2012). Jalal, on the contrary, has noted that almost all of his stories were written when he was sober and therefore credits this prolific output with the period of sobriety that lasted for some months after his first hospital admission (2013). Regardless, it is clear that Manto knew his drinking was killing him. He wrote in 1954 that ‘anyone drinking this dreadful stuff ends up in the netherworld in less than a year’ (Jalal 2013, 191). According to his nephew Hamid Jalal, Manto might have been ‘toying with the idea of suicide, either because it was the easiest way out or because he wanted to fill the family with remorse for having given him up as hopeless case’ (192). Whether or not his drinking amounted to a form of “extended suicide” it is clear that Manto’s battle with addiction would have influenced his work.

## Mental illness as a theme in “Toba Tek Singh”

Mental illness is an important and enduring, perhaps even defining, theme in “Toba Tek Singh.” Indeed the choice to write about partition through the lens of a mental asylum is itself highly significant. Manto’s use of the patients to reflect the “madness” of what was happening outside was poignant. The asylum in a sense represents the whole subcontinent (Ispahani 1988); the madness of its inhabitants symbolising the madness of the partition violence. Bishan Singh’s nonsense phrases, as Tarun K. Saint has explained, reflect the arbitrariness and opacity of the governmental machinery (2012). Increasingly, it becomes clear that the “lunatics” in the asylum are more sane than the government figures making decisions about their exchange. Astute comments by the asylum inmates demonstrate the absurdity of partition:

All those lunatics in the asylum who had at least some sense left were uncertain whether they were in Pakistan or India. If they were in India, then where was Pakistan? If they were in Pakistan, how could it be possible when only a short while ago they had been in India, without having moved at all? (Manto 1955, 9)

“Toba Tek Singh” was written after Manto’s time in hospital and was clearly influenced by his experience; perhaps even the choice to write about a mental asylum was a consequence of his hospitalisation. In a context where mental illness was commonly regarded as abhorrent and shameful, Manto’s explicit engagement with the theme would have been unusual but powerful, emphatically drawing attention to the madness of partition.

The character of Bishan Singh represents a symbolic commentary on the trauma of displacement. His intense suffering reflects that of partition refugees. The constant questioning and demands to know about his homeland are evocative of fractured identities and loss of sense of belonging. Perhaps his character is also a reflection of Manto’s own suffering and confusion about identity in the wake of his move to Lahore. Manto wrote: ‘I found my thoughts scattered. Though I tried hard, I could not separate India from Pakistan and Pakistan from India’ (Hasan 1984, 89). Elsewhere he added: ‘I found it impossible to decide which of the two countries was now my homeland’ (Ispahani 1988,192). In this way, Bishan Singh’s character can be read both as a mirror to the general displacement suffered by so many as well as a more specific portrayal of Manto’s own personal experience. From both perspectives, the pain and emotional trauma of displacement is significant, often contributing to psychopathology whether implicitly or explicitly.

## Context and implications

It is crucial to take into account the specific historical and geographical context in which Manto wrote, within which the underlying influences and subsequent implications of his work should be situated. The exchange of psychiatric inpatients between India and Pakistan actually happened, as described by Sanjeev Jain and Alok Sarin. After 1947, the partition of the Punjab Mental Hospital in Lahore dragged out over several years as there was nowhere to transfer patients. In 1949, the Amritsar asylum was hastily constructed, which although inadequate, was able to receive four hundred fifty non-Muslim patients in 1950. Of those, two hundred eighty-two were kept there with the remainder being sent on to Ranchi. Two hundred thirty-three Muslim patients, meanwhile, were sent to Lahore from various Indian hospitals. Patients were largely classified based on who would pay the bills (Jain and Sarin 2012). This almost complete disregard for identity and individuality, where the mentally ill were treated as merely an administrative burden, was typical of the time.

Manto’s work was never moralising; he left it up to his readers to form judgements, preferring instead to record events bluntly without comment (Jalal 2013). However, an engagement with, and indeed challenge towards, popular attitudes is implicit in “Toba Tek Singh.” Manto’s blunt and matter-of-fact descriptive style presents mental illness as a fact of life; it is neither dramatised nor evaded, nor is it trivialised as cheap comedy. It is just there. This was a normalising approach. Significantly there is a conspicuous absence of psychiatrists in “Toba Tek Singh”; the target of Manto’s criticism, as Saint has argued, was not mental health professionals or the practice of psychiatry but rather the bureaucratic procedures and those according to whose whims they were implemented (2012). The publication of the story may itself have had some impact on the exclusionist popular attitudes towards the mentally ill that were so prevalent at the time. Jain and Sarin have described how the Amritsar asylum psychiatrist, Dr. Vidysagar, by accommodating patients and their families together in tents due to the inadequacy of facilities, pioneered a model for greater family engagement in the care of the mentally ill (2012). It is conceivable that Manto’s normalising writings may have contributed, if only in a limited way, to the beginnings of a shift in popular opinion.

The implications of Manto’s work have been touched on above but were more concrete with respect to his literary influence. “Toba Tek Singh” was not the only one of his stories to deal with mental illness. Indeed, as Stephen Alter has argued, ‘in the period following Partition, madness became the guiding metaphor in much of Manto’s fiction’ (Alter 1994, 96). For example, in “Khol Do,” Manto’s use of the character Sakinah’s dissociative state following the trauma of rape is shocking and powerful. Mental illness as a literary theme has been used by a number of writers working on partition long after Manto’s death; in this sense his work on mental illness cast a ‘long shadow’ (Saint 2012, 59). Indeed, as Alter has described, madness became the ‘only conceivable response’ to the ‘ruthless inhumanity of Hindu-Muslim violence’ (1994, 91). As Saint has explained, partition violence meant huge psychological trauma, that often manifested in belated after-effects. Whole communities were affected by symptoms of post-traumatic stress disorder. The literature on partition and mental illness, then, may have helped to enable the beginnings of a process of working through these traumatic memories (2012). This was, and continues to be, a long and difficult process, but one of the most important implications of Manto’s work was to help set this in motion.

## Conclusion

Manto’s writing in general, and “Toba Tek Singh” in particular, was coloured by his own mental health problems, namely alcohol addiction and possibly depression. Even the choice to use a mental asylum to reflect the “madness” of partition was intimately related to his experience. However, more than just this, “Toba Tek Singh” and the character of Bishan Singh is a symbolic commentary on the psychological trauma of the human displacement brought about by partition; perhaps also the author’s own displacement and uncertainty about identity. The specific subcontinental context was important in terms of attitudes towards, and treatment of, the mentally ill around the time of partition. Importantly, Manto’s work started a trend of writing about mental illness and partition; later authors followed suit. This may have helped audiences to go some way towards processing their psychological trauma. Although this analysis has focused specifically on “Toba Tek Singh,” references to mental illness and psychological distress are prevalent in many of Manto’s other stories, “Khol Do” being just one example. The broad range of Manto’s corpus of work now needs to be examined from this angle in order to shed further light on the relationship between his literature and mental illness in the Indian subcontinent.
